# Mucin Deficiency Causes Functional and Structural Changes of the Ocular Surface

**DOI:** 10.1371/journal.pone.0050704

**Published:** 2012-12-18

**Authors:** Anne M. Floyd, Xu Zhou, Christopher Evans, Olivia J. Rompala, Lingxiang Zhu, Mingwu Wang, Yin Chen

**Affiliations:** 1 Department of Pharmacology and Toxicology, University of Arizona, Tucson, Arizona, United States of America; 2 Department of Ophthalmology and Vision Science, College of Medicine, University of Arizona, Tucson, Arizona, United States of America; 3 Department of Ophthalmology, Fourth Affiliated Hospital, China Medical University, Shenyang, China; 4 Department of Medicine, University of Colorado, Aurora, Colorado, United States of America; Wayne State University, United States of America

## Abstract

MUC5AC is the most abundant gel-forming mucin in the ocular system. However, the specific function is unknown. In the present study, a *Muc5ac* knockout (KO) mouse model was subject to various physiological measurements as compared to its wide-type (WT) control. Interestingly, when KO mice were compared to WT mice, the mean tear break up time (TBUT) values were significantly lower and corneal fluorescein staining scores were significantly higher. But the tear volume was not changed. Despite the lack of *Muc5ac* expression in the conjunctiva of KO mice, *Muc5b* expression was significantly increased in these mice. Corneal opacification, varying in location and severity, was found in a few KO mice but not in WT mice. The present results suggest a significant difference in the quality, but not the quantity, of tear fluid in the KO mice compared to WT mice. Dry eye disease is multifactorial and therefore further evaluation of the varying components of the tear film, lacrimal unit and corneal structure of these KO mice may help elucidate the role of mucins in dry eye disease. Because *Muc5ac* knockout mice have clinical features of dry eye, this mouse model will be extremely useful for further studies regarding the pathophysiology of the ocular surface in dry eye in humans.

## Introduction

Mucus, a viscoelastic and gel-like substance, covers the mammalian epithelial surface of various tissues including the ocular, respiratory, digestive, and reproductive systems. Other than acting as a passive barrier, mucus has many important functions in regulating epithelial homeostasis and innate mucosal defenses. The major macromolecular components of mucus are high-molecular-weight mucin glycoproteins. The viscous and elastic properties of the mucus gel have been suggested to be largely caused by the physical properties and structural features of mucin glycoproteins [1]. To date, at least twenty four genes have been designated “*MUC*” (human gene) or “*Muc*” (mouse gene), and include *MUC1-2, 3A, 3B, 4, 5AC, 5B, 6–21, and 24.* (http://www.ncbi.nlm.nih.gov/gene). Mucin family is very heterogeneous. Based on structural and functional features, mucins have been grouped into three categories; membrane-bound mucins, large gel-forming mucins, and soluble mucins [2]. *MUC2, MUC5AC, MUC5B, MUC6 and MUC19* define a gel-forming mucin subfamily. Gel-forming mucins are believed to be evolved from the common ancestor, von Willebrand factor (vWF) [3]. The gel-forming mucins are of large size (15 kb–40 kb cDNA) and share similar structural and sequence features, including multiple “cysteine-rich” von Willebrand factor D- or C-like domains (VWD, VWC), a long central region with multiple threonine/serine rich repeats (sites of oligosaccharide attachment) and a C-terminal cystine knot (CT) domain [3], [4]. The number and position of cysteines within VWD, VWC and CT domains are extremely conserved. For example, eleven cysteine residues in the CT domain are conserved across the gel-forming mucins and vWF. The cysteine-rich domains appear to play essential roles in forming disulfide- linked dimers [5], [6] and multimers [3], [7], [8]. No such domains are found in other mucins. The large size, extended structure and formation of multimers via covalent disulfide bonds suggest a pivotal role for gel-forming mucins in forming the mucus gel. Alterations in the expression of gel-forming mucins can directly affect the composition and physiological properties of mucus and airway homeostasis, as implicated in various chronic airway diseases, cancer, etc [9]–[11].

A normal tear film (TF) is required to maintain the health and function of the ocular surface. TF maintains a smooth ocular surface for normal vision, protects from infections and environmental hazards, and maintains ocular comfort and a healthy epithelium. In eye, gel-forming mucins act as a surfactant for the ocular surface, allowing an evenly spread TF to wet the hydrophobic epithelium [12]. They are thought to be responsible for epithelium protection, maintenance of optical purity and refractive power [12]. The concentration of mucins in TF increases toward the ocular surface. Conjunctival goblet cells are responsible for the production of the gel-forming mucins [12]. Among all the gel-forming mucins, MUC2, MUC5AC and MUC19 have been detected in human conjunctival tissue and MUC5AC appears to be the most abundant gel-forming mucin in the ocular system [13]–[15]. However, the specific function of MUC5AC is largely speculated, but not experimentally defined. In the present study, we take advantage of the recently available deficient mouse model to investigate the function of Muc5ac in the ocular system.

## Materials and Methods

### 1. Creation of Muc5ac-deficient Mouse by Targeted Gene Mutation and Use of the Animal

The creation of Muc5ac deficient mouse was described in details elsewhere [16]. Briefly, the Muc5ac locus was targeted by inserting LoxP sites into the 5′-flanking region and intron 1 in CJ7 embryonic stem cells. Global knockout mice were then produced by mating founder animals with Zp3-Cre transgenic (C57BL/6-Tg(Zp3-cre)93Knw/J) and subsequently crossing progeny with C57BL/6J mice. Mice were backcrossed onto a C57BL/6J lineage for ten generations, and saturation of the C57BL/6J genome was confirmed using microsatellite marker–assisted congenic analysis at the University of Texas MD Anderson Cancer Center Genetic Services Facility. After transferred to animal facility of University of Arizona, the mice were housed in a standard environment during the study as follows: room temperature 71°F, relative humidity 46±2%, and alternating light-dark cycles (7 am to 7 pm). DNA extracted from mouse tail biopsies was screened by long-range PCR to identify Muc5ac WT (+/+), heterozygous (+/−), and knockout (−/−) animals. Prior to evaluation, the mice were immobilized with an intraperitoneal injection of Avertin [0.25–0.50 mg/g]. This study was conducted in compliance with the Tenets of the Declaration of Helsinki and ARVO statement for the Use of Animals in Ophthalmic and Visual Research.

### 2. Tissue RNA Extraction, RT-PCR and Realtime PCR

Both wild-type and knockout mice were sacrificed and their ocular tissues (cornea, conjunctiva, lacrimal gland) were dissected. Total RNAs were extracted and RT-PCR was performed as described previously [17]. Briefly, cDNA was prepared from 3 µg of total RNA with Moloney murine leukemia virus (MoMLV)–reverse transcriptase (Promega. Madison, MI) by oligo-dT primers for 90 min at 42°C in a 20-µl reaction solution, and PCR was performed to detect *Muc5ac* expression. Housekeeping gene *Actin* was used as an internal control. Real-time PCR was performed as described previously [18]. Two microliters of diluted cDNA was analyzed using 2x SYBR Green PCR Master Mix by an ABI 5700 or ABI Prism 7900HT Sequence Detection System (Applied Biosystems. Foster City, CA), following the manufacturer’s protocol. Primers were used at 0.2 µM. The P CR reaction was performed in 96-well optical reaction plates, and each well contained a 50-µl reaction mixture. The SYBR green dye was measured at 530 nm during the extension phase. The relative amount of mRNA in each sample was calculated based on the Δ**Δ**C_t_ method using housekeeping gene *GAPDH*. The purity of amplified product was determined from a single peak of a dissociation curve. Efficiency curves were performed for each gene of interest relative to the housekeeping gene, based on the manufacturer’s instructions. Primer sequences are listed in Table 1.

**Table 1:** Primers.

Gene	Primers	
*Muc5ac* (regular)	forward	CTGATAGTCACATACGAGGAGGG
	reverse	TCTTGGGACACTCTGTCTTCATT
*Actin* (regular)	forward	AGCCATGTACGTAGCCATCC
	reverse	TAGAAGCACTTGCGGTGCAC
*Muc1* (realtime)	forward	TGGGGATCTCTAGCATCAAGTT
	reverse	GCTCCTACAAGTTGGCAGAAGT
*Muc2* (realtime)	forward	GTGCTGCAATATCACCTCATGT
	reverse	TGTATGTGATGGAGCCTGAAAC
*Muc4* (realtime)	forward	CGGGATTCCTTCTACGTTACAG
	reverse	GAATCGATCTGGACGGTACTTC
*Muc5ac* (realtime)	forward	CCATGCAGAGTCCTCAGAACAA
	reverse	TTACTGGAAAGGCCCAAGCA
*Muc5b* (realtime)	forward	GCTGCTGTTACTCCTGTGAAAAAG
	reverse	TGACCTCTGTCTCACAGCCCTTA
*Actin*(realtime)	forward	ACCGTGAAAAGATGACCCAGA
	reverse	GGAGTCCATCACAATGCCTGT

### 3. Mucous Cells Staining by Either Alcian Blue/PAS or Mucin Antibodies

AB/PAS staining was used to detect mucous (goblet) cells as described previously [19]. Alcian blue stains acidic mucins and PAS stains neutral mucins. Briefly, slides were first stained with Alcian blue for acidic mucins, and washed out. Then, the slides were treated with periodic acid, washed in distilled water and then stained with Schiff’s reagent. To assist with the morphological analysis, H&E staining on the serial section of the same slide were used as comparisons. For specific mucin staining, monoclonal anti-Muc5ac (45M1, Thermo Fischer Scientific. Kalamazoo, MI) and polyclonal anti-Muc5b (H300, Santa Cruz Biotechnology. Santa Cruz, CA) were used. For dual staining, anti-mouse IgG conjugated with Alexa 488 and anti-rabbit IgG conjugated with Alexa 594 (Invitrogen. Grand Island, NY) were used sequentially. For morphometric analysis, the staining area and the basement membrane length were automatically calculated, and the results were presented as (staining positive area/base membrane length). 3 sections per mouse and 5 mice each (for either WT or KO) were counted.

### 4. Fluorescein Staining

Fluorescein staining was performed based on the protocols [20], [21] described previously with some modifications. Liquid sodium fluorescein [1 µL, 0.25%] was placed in the conjunctival sac. After 1 minute of exposure, the excess fluorescein was rinsed with 4 drops [0.2 mL] of HBSS and blotted with Q-tips. Fluorescein staining was analyzed with a cobalt blue light under a dissecting microscope. The scores are: 0 = absent staining; 1 = slightly punctate staining <30 spots; 2 = punctate staining >30 spots but not diffuse; 3 = severe diffuse staining but no positive plaque; 4 = positive fluorescein plaque. Representative photographs were taken with a consumer grade SLR camera [Canon Rebel] connected to a dissecting microscope [Olympus, Japan] via a microscope adaptor.

### 5. Tear Breakup Time (TBUT) Assay

The similar protocols [20], [21] were followed except that the examiners were masked for this evaluation. Liquid fluorescein was applied as described above. After 3 blinks, tear breakup time was recorded in seconds with a cobalt blue filter under a dissecting microscope. The final TBUT was an average of 3 separate measurements.

### 6. Tear Volume Test

Tear volume was determined with the phenol red thread tear test [ZoneQuick; FCI Ophthalmics] [22]. The lower eyelid was slightly lowered to place a 1 mm portion of the thread on the palpebral conjunctiva at approximately 1/3 of the distance from the lateral canthus of the lower eyelid. Each eye was individually tested with the eye open for 15 seconds. The red portion of the thread was recorded in millimeters. The final length was an average of 3 separate measurements.

### 7. Statistical Analysis

Statistical analysis was performed with SPSS 19 (SPSS, Chicago, IL). Means, standard deviations, Statistics Fisher’s Exact Test and independent t-tests were applied in all comparisons between groups. p-values are stated with p<0.05 considered statistically significant.

## Results

### 1. The Lack of *muc5ac* Expression in the Ocular Tissues of *Muc5ac* Deficient (KO) Mice

**Figure 1:**
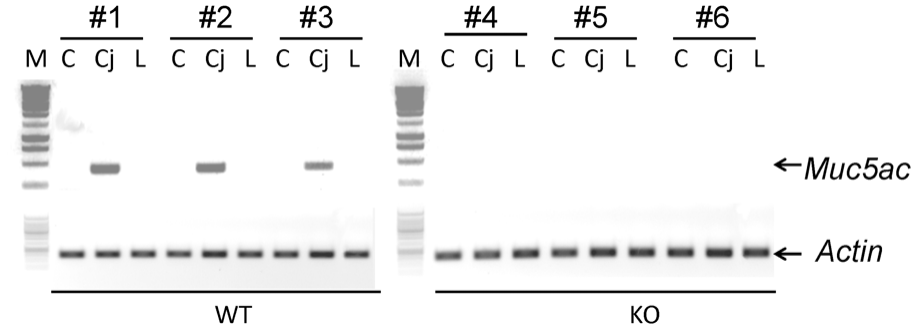
Lack of *Muc5ac* in the ocular tissues of *Muc5ac* knockout mice. Cornea (C), Conjunctiva (Cj), and Lacrimal gland (L) were dissected and total RNA was extracted for RT-PCR assay. M: molecular weight marker. *Actin* was used as a loading control. #1, 2, 3, 4, 5, 6 were different mice. WT: wild-type mice. KO: *Muc5ac* knockout mice.

Three major ocular tissues (cornea, conjunctiva, and lacrimal gland) were dissected. Total RNA was collected and subject to RT-PCR analysis. Consistent with the literatures [13], [14], [15], conjuctiva had abundant *Muc5ac* gene expression in the wide-type (WT) mice (Fig. 1). No *Muc5ac* expression was detected in the cornea and the lacrimal gland. In contrast to the WT mice, KO mice had no *Muc5ac* expression in any of these tissues (Fig. 1).

### 2. Compensatory Increase of Muc5b was Detectd in *Muc5ac* Deficient (KO) Mice

**Figure 2:**
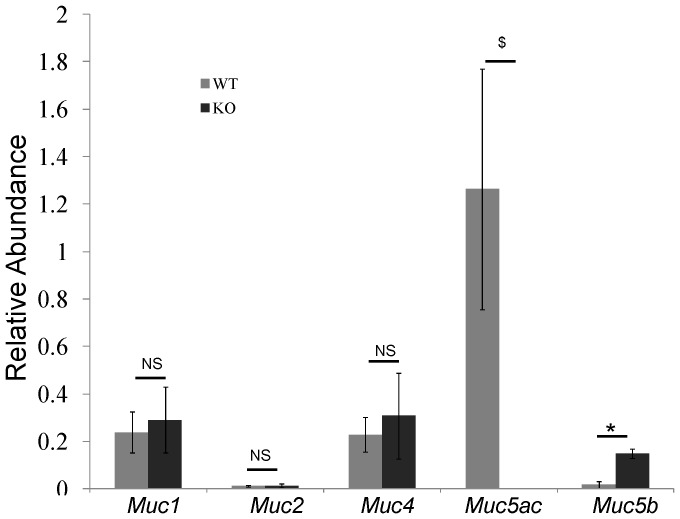
Mucin gene expressions in the conjunctiva tissues of wild-type and *Muc5ac* knockout mice. Conjunctiva was dissected and total RNA was extracted for realtime PCR assay. n = 5, $, *: p<0.05. NS: not significant. WT: wild-type mice. KO: *Muc5ac* knockout mice.

**Figure 3:**
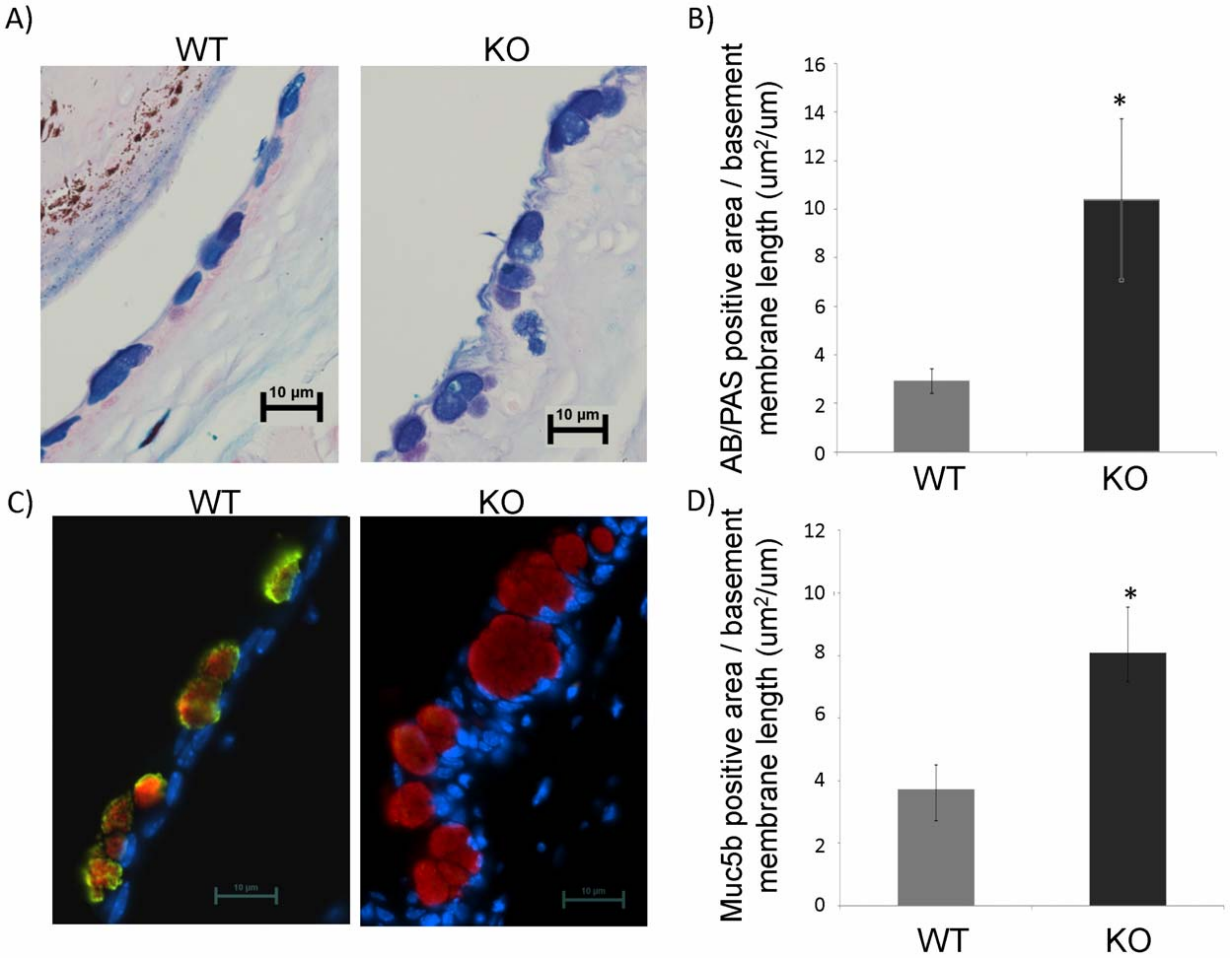
Significantly increased mucous cell staining positive areas. Mouse conjunctiva tissues were harvested, fixed and the tissue sections were stained with AB/PAS or anti-Muc5ac and anti-Muc5b antibodies. A) Representative images (60X, 10 um scale bar was shown) from AB/PAS staining on mouse conjunctiva tissues. B) Statistical analysis of AB/PAS staining positive area per basement membrane length. n = 5, *: p<0.05. C) Representative images (60X, 10 um scale bar was shown) from dual staining of Muc5ac and Muc5b on mouse conjunctiva tissues. D) Statistical analysis of Muc5b staining positive area per basement membrane length. n = 5, *: p<0.05. WT: wild-type mice. KO: *Muc5ac* knockout mice.

Because Muc5ac is the major mucin in conjunctive mucous (goblet) cells, we tested whether or not there is a compensatory increase of other mucin gene in KO mice. Among *Muc1, 2, 4* and *5b*, only *Muc5b* had a significant increase in KO mice (Fig. 2). Then, we tested if mucous cells were changed due to the lack of Muc5ac protein. AB/PAS staining was used to detect mucous cells. Consistent with the literature [12], mucous cells were present abundantly on the surface of conjunctiva (Fig. 3A). In addition, as compared to WT mice, these mucous cells were significantly larger (Fig. 3A). Due to the low resolution of the AB/PAS staining, some cells appeared to be fused together, which makes it difficult to count the cell number precisely. Thus, we chose instead to quantify these cells by measuring the AB/PAS staining positive area per basement membrane length. Indeed, the positively staining areas were markedly increased in KO as compared to control (Fig. 3B), suggesting a potential increase of mucous glycoproteins in these cells. Next, we examined the protein expressions of Muc5ac and Muc5b using specific antibodies. In WT mice, almost all mucous cells expressed both Muc5ac and Muc5b (Fig. 3C). As expected, there was no Muc5ac staining on KO mice (Fig. 3C). But, Muc5b staining positive areas were significantly increased as compared to WT mice (Fig. 3C–D), suggesting the increase of Muc5b protein. This may count for the increase of mucous content in these cells (Fig. 3A–B).

### 3. Structural Changes in a Small Percentage of *Muc5ac* Deficient (KO) Mice

**Figure 4:**
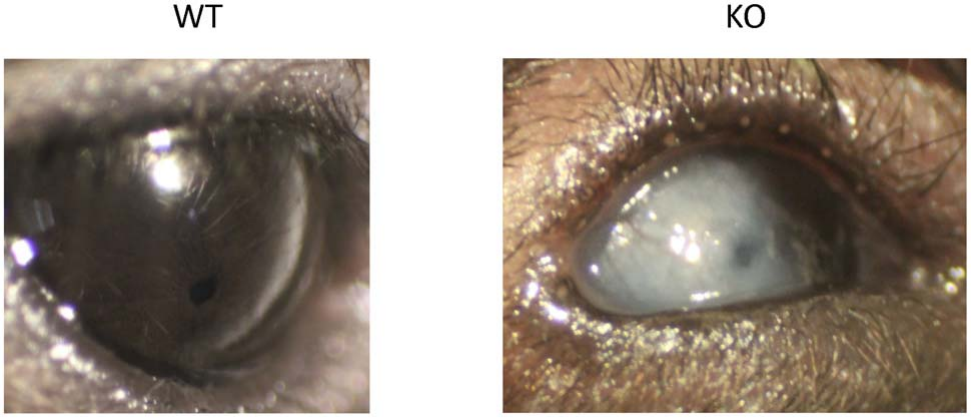
Representative photographs of corneal opacification are shown. Corneal opacification of various degrees was noted in both eyes of 4 out of 35 Muc5ac-deficient mice (KO), but not in any of the wild-type mice (WT).

Although most of the KO mice had no obvious ocular abnormality, 4 mice (out of total 35 mice, or 11.4%) developed corneal opacification of varying severity and location. For instance, one mouse developed a severe, unilateral, full thickness corneal opacification with neovascularization (Fig. 4). This structural change usually started from one eye and later progressed into the other eye. This phenomenon has never been observed among our wild-type controls or other C57BL/6J mice, nor has it been reported in the literature as an occurrence in the C57BL/6J mice.

### 4. Evaluation of the Ocular Surface of *Muc5ac* Deficient (KO) Mice

**Figure 5:**
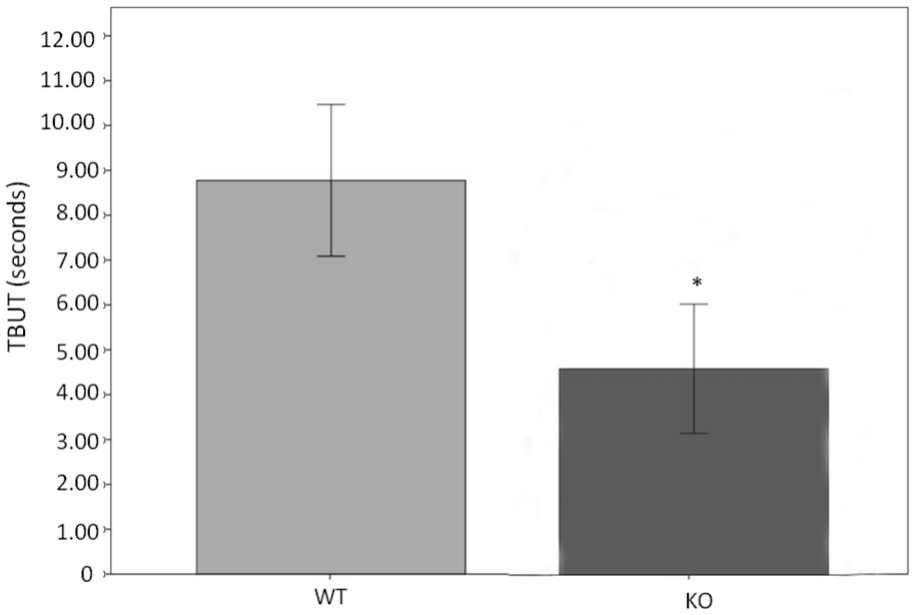
Tear stability is decreased in *Muc5ac*-deficient mice (KO) compared to wild-type mice (WT). This is determined by measuring tear film breakup time (TBUT). Bar graph shows the mean TBUT in KO and WT. TBUT values were obtained in a masked fashion under a cobalt blue filter after the application of fluorescein dye. Three measurements were averaged to generate the final value, which represent the data presented. Total 31 KO and 15 WT were compared. These two groups were gender and age matched. (*: p = 0.001, independent t-test, error bars indicate 95%CI).

**Figure 6:**
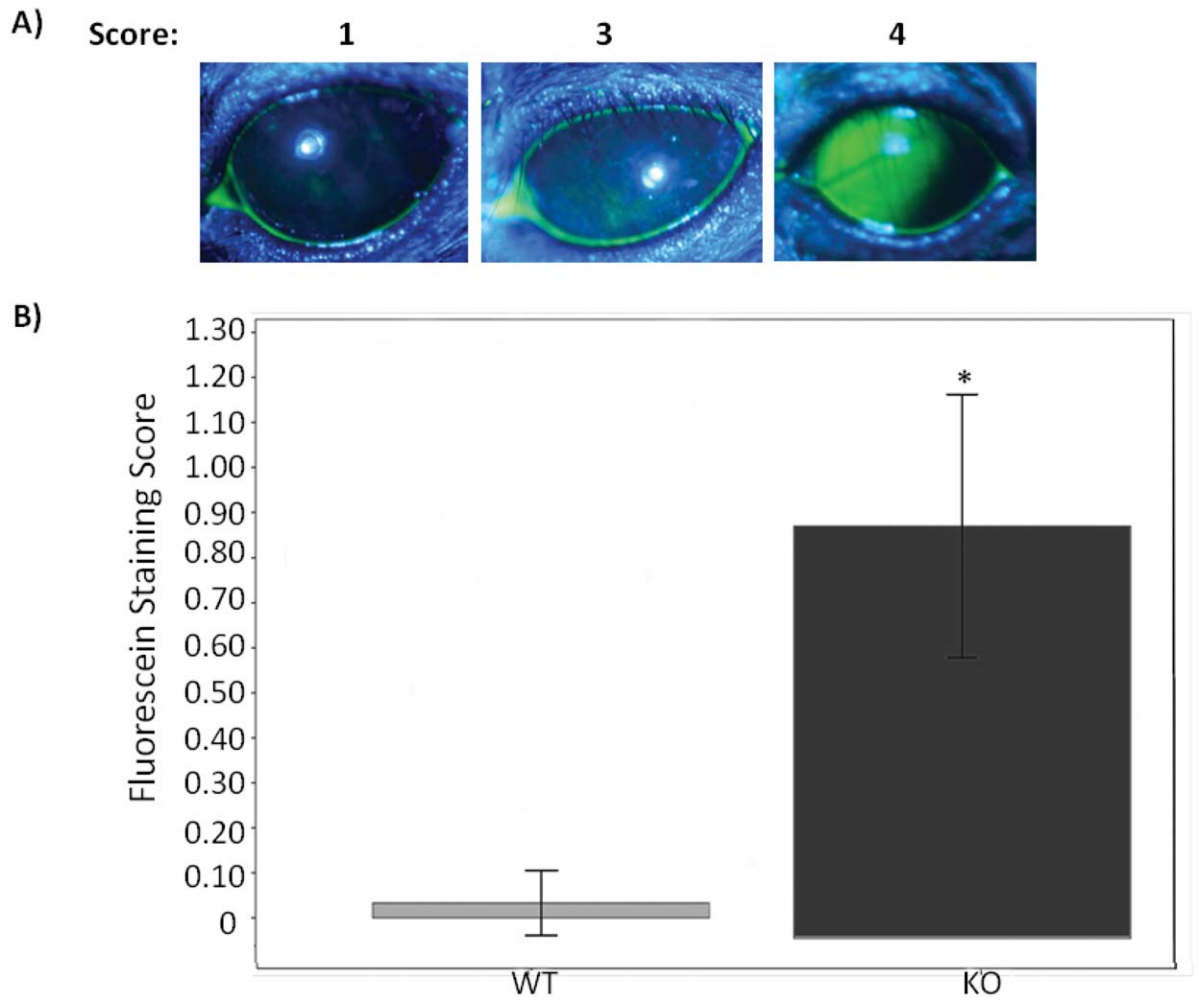
Corneal epithelium is disrupted in Muc5ac-deficient mice (KO) compared to wild-type mice (WT). This is determined by corneal fluorescein staining. Liquid fluorescein (1 ul, 0.25%) was applied for 1 minute and the excess fluorescein was rinsed with 0.2 mL of HBSS and blotted with q-tips. The fluorescein staining was analyzed under a cobalt blue filter and scored as described in the method section. Eyes were closed between each evaluation to prevent excessive exposure and irritation of ocular surface. A) Representative photographs of fluorescein staining demonstrate the corneal staining scores (scores 1, 3, and 4 are shown). B) Bar graph shows the mean score for corneal staining in KO and WT. The mean fluorescein scores from total 31 KO and 15 WT were compared. These two groups were gender and age matched. (*: p<0.001, independent t-test, error bars indicate 95%CI).

**Figure 7:**
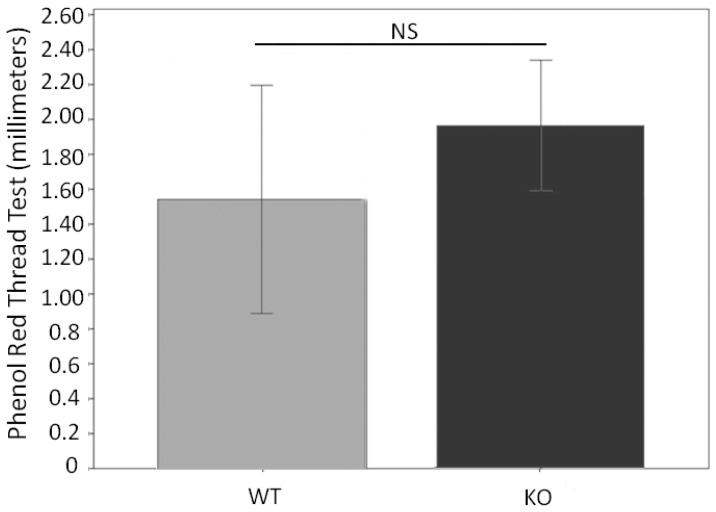
Tear volume is similar in Muc5ac-deficient mice (KO) compared to wild-type mice (WT). This is determined by red phenol thread test. Bar graph shows the mean thread measurement in *Muc5ac*-deficient mice and wild-type. Three measurements (mm) were obtained with the thread placed in the lower lid for 15 seconds with the eyes open. These 3 values were averaged to generate the final measurement, representing the data presented. The mean values from total 31 KO and 15 WT were compared. These two groups were gender and age matched. (NS: Not statistically significant. p = 0.061, independent t-test, error bars indicate 95%CI).

Since all the other mice appear to be normal, we performed several ocular surface tests. We first tested tear stability by tear film breakup time (TBUT) assay. There was a significant decrease of TBUT in KO mice compared to WT mice (Fig. 5), suggesting the instability or incompleteness of the tear film. To corroborate this result, we performed fluorescein staining on the ocular surface. The hypothesis to be tested here was that, if there was indeed an unstable tear film, ocular surface would be stained. Indeed, KO mice had a dramatic staining on the ocular surface as compared to the WT mice which had essentially very little staining (Fig. 6A–B). Interestingly, the total tear volume was not significantly different between KO and WT mice (Fig. 7). Overall, These results demonstrate a decline in the quality of the tears without altering the tear quantity.

## Discussion

Knockout mouse is perhaps the best model to determine gene function in a physiologically-relevant setting. The first gel-forming mucin deficient model is *Muc2* knockout mouse. Since Muc2 is the dominant mucin in small intestine and colon, these mice displayed significant pathological phenotypes, such as inflammation [23], aberrant intestinal crypt morphology and altered cell maturation and migration [24]. They spontaneously developed colitis [23], and also frequently developed adenomas in the small intestine that progressed to invasive adenocarcinoma, as well as rectal tumors [24]. These results suggest that dominant gel-forming-Muc2 plays a critical role in the maintenance of epithelial homeostasis in small intestine and colon. Recently, Hasnain SZ et al. have shown that Muc5ac protein is a critical component in the defense against intestinal nematode infection using a *Muc5ac* deficient mouse model [16].

Since Muc5ac is the dominant gel-forming mucin in the ocular system, we take advantage of this model to evaluate the potential role of Muc5ac in ocular surface. Although we couldn’t obtain the accurate mucous cell count due to the low resolution of the AB/PAS staining, significant increase of mucous cell areas were observed in KO mice as compared to the control. This is consistent to the compensatory increase of the mRNA and protein of *Muc5b*, another major gel-forming mucin [3]. Previously, Wang et.al used a *Muc5ac*-promoter driven DTA to deplete conjunctival goblet cells [25]. But, they didn’t find any alteration on ocular surface. Although the increase of *Muc4* was thought to compensate the decrease of *Muc5ac*, significant amount of *Muc5ac* (more than half) remained in that model, which may be sufficient to maintain the normal function. In the present study, despite the enlargement of mucous cells (containing more mucous glycoprotein) and significant compensatory increase of Muc5b, we have found that the lack of *Muc5ac* markedly destabilizes the tear film (TF). This observation has demonstrated, for the first time, the indispensable role of *Muc5ac* in the maintenance of ocular hemostasis. A normal TF is maintained by the lacrimal glands, ocular surface (cornea and conjunctiva), Meibomian glands, lids, and their innervations [26]. Together as a functional unit, they control secretion of the three major components of the TF (i.e. aqueous, lipid, and mucin) [27]. Our finding suggests that the dysfunction of the mucin component is sufficient to cause TF instability or alter its composition so that it no longer supports the normal function of the ocular surface. Although we demonstrate the intact tear volume, we haven’t had a chance to investigate the lipid component, which requires further study.

One of major ocular diseases that affect mucin expression is the dry eye syndrome. Dry eye disease affects nearly 30% of Americans causing discomfort and impaired vision, which interferes with daily activities. The pathogenesis of dry eye is multifactorial, but ultimately is a consequence of quantitative and/or qualitative TF insufficiency [14]. Muc5ac has been shown by both us [15] and others [12], [28] to be downregulated in the dry eye from Sjogren syndrome. But the causality has not been established. By using a state-of-the-art *Muc5ac* deficient model, we, for the first time, demonstrate the causal link between the lack of *Muc5ac* and the qualitative TF insufficiency. This finding suggests that artificial tear (or eye drop) modulating ocular surface mucin may have significant beneficial effect on dry eye patient. Indeed, the therapies under clinical trial include the compounds stimulating mucin secretion (e.g., rebamipide, an amino acid analogue of quinolinone), and restoring mucins on the ocular surface (e.g. gefarnate, a water-insoluble terpene fatty acid) [29]. In addition, some over-the counter artificial tears use high-molecular-weight compound to emulate mucin properties (e.g. Optive®). Most recently, Konat Zorzi et, al. has carried out a proof-of-concept experiment [30], in which nanoparticles are used to deliver MUC5AC expression plasmid into ocular surface epithelial cells to treat dry eye syndrome. Therefore, thorough understanding of the biological effect of mucin on the ocular surface will facilitate the improvement of these medications to treat dry eye syndrome.

Most notably, a few KO mice have developed opacification on their ocular surface (mainly on cornea). Although the number is small (11.4%), none of the similar observation has been found in WT mice. C57BL/6J is a widely-used mouse strain. We have never made such observation in other C57BL/6J mice, and there is no literature reporting ocular opacification in these mice. Thus, this abnormality may well be associated with the lack of *Muc5ac*. But, considering the small percentage, other unknown causes might also be involved. It is well documented that corneal opacification may be caused by environmental [31]and genetic [31], [32] factors. Viral infection (most commonly herpes virus) and physical injury are the two most common environmental factors. Limbal stem cell deficiency has been implicated in the corneal opacification caused by these environmental insults [33]. Thus, the low percentage of our observation may reflect the opportunistic injury or infection that occurs in certain occasion. Through the study on congenital corneal opacification, dozens of susceptible genes have been identified [32]. These genes have diverse functions, suggesting the opacification may be controlled by multiple genes [32]. Thus, it is also possible that *Muc5ac* may just be one of these susceptible genes, and its deficiency has a low penetration. Nonetheless, this observation needs further investigation.

In summary, we have found significant structural and functional changes of the ocular surface in the *Muc5ac* deficient mouse model. Complete understanding of mucin function in ocular system will advance our knowledge in this area and facilitate the development of effective medication treating dry eye syndrome.
